# Barriers to prescribing and insurance approval of SGLT2 inhibitors for heart failure: a mixed-methods study in Jordan

**DOI:** 10.3389/fphar.2026.1803731

**Published:** 2026-04-07

**Authors:** Zekrayat J. H. Merdas, Anas Abed, Mohammad Abu Assab, Wael Abu Dayyih, Mona Bustami, Leen M. Saudi, Sireen Abdul Rahim Shilbayeh

**Affiliations:** 1 Pharmacy Department, College of Pharmacy, Amman Arab University, Amman, Jordan; 2 Department of Biopharmaceutics and Clinical Pharmacy, Faculty of Pharmacy, Al-Ahliyya Amman University, Amman, Jordan; 3 Clinical Pharmacy Department, Faculty of Pharmacy, Zarqa University, Zarqa, Jordan; 4 Faculty of Pharmacy, Mutah University, Alkarak, Jordan; 5 Faculty of Pharmacy, University of Petra, Amman, Jordan; 6 Department of Pharmacy Practice, Princess Nourah bint Abdulrahman University, Riyadh, Saudi Arabia

**Keywords:** heart failure, insurance approval, Jordan, misclassification of indications, mixed-methods study, SGLT2 inhibitors

## Abstract

**Background:**

Sodium–glucose cotransporter 2 inhibitors are now cornerstone therapy for heart failure across the ejection fraction spectrum, independent of diabetes status. Despite strong guideline recommendations, real-world uptake remains suboptimal, especially in middle-income settings. Evidence on the interplay between prescribing decisions and insurance authorization processes is limited.

**Objectives:**

To explore knowledge, attitudes, prescribing practices, and authorization decision-making related to SGLT2 inhibitors for HF among treating physicians and insurance physicians in Jordan, and to identify system-level barriers to evidence-based use.

**Methods:**

An explanatory sequential mixed-methods design was used. A cross-sectional survey was conducted among treating physicians (general practitioners, family medicine, internal medicine) and insurance physicians involved in medication authorization. The validated questionnaire was analyzed with descriptive statistics and multivariable logistic regression. Semi-structured interviews followed with a purposive sample; reflexive thematic analysis was applied. Findings were integrated using joint displays.

**Results:**

Of 312 physicians surveyed (214 treating, 98 insurance), treating physicians showed moderate-to-good knowledge and favorable attitudes toward SGLT2 inhibitors in HF, yet initiation rates were low. Consistent insurance approval for HF without diabetes occurred in fewer than one-third of cases. Misclassification of SGLT2 inhibitors as diabetes-only medications was prevalent among insurance physicians (61.2%) and the strongest predictor of rejection (adjusted OR 0.18, 95% CI 0.10–0.33). Lack of guideline-aligned protocols and non-cardiologist prescriber status further reduced approval. Qualitative data highlighted drug-class identity, professional hierarchies, cost accountability, and defensive decision-making as key influences. Integrated findings showed system-level factors overriding physician knowledge and intent.

**Conclusion:**

Barriers to SGLT2 inhibitor use for HF in Jordan are predominantly systemic. Misclassification and misalignment between evidence and insurance frameworks hinder guideline-directed care. Policy interventions, updating reimbursement structures, standardizing criteria, and involving stakeholders, are essential to improve access.

## 1 Introduction

Heart failure (HF) remains a major public health challenge worldwide, with increasing prevalence, high rates of hospitalization, and substantial morbidity and mortality despite advances in pharmacotherapy (Shahim et al., 2023). The burden of HF is particularly pronounced in low- and middle-income countries, where healthcare systems face additional challenges related to access, cost containment, and fragmented models of care (Mbanze et al., 2025). In Jordan, HF represents a growing clinical and economic burden, driven by population aging, high rates of diabetes mellitus and hypertension, and variability in access to specialized cardiovascular services (Al-Sutari and Abdalrahim, 2024; Abu-Hantash et al., 2025; Al-Ajlouni et al., 2024).

Over the past decade, sodium–glucose cotransporter 2 inhibitors (SGLT2 inhibitors) have emerged as a transformative therapeutic class in the management of HF (Crispino et al., 2025). Initially developed as glucose-lowering agents for type 2 diabetes mellitus, large randomized controlled trials have consistently demonstrated that SGLT2 inhibitors significantly reduce heart failure hospitalizations and cardiovascular mortality in patients with heart failure with reduced ejection fraction (HFrEF), irrespective of diabetes status (de Souza et al., 2025). Large randomized controlled trials have provided robust quantitative evidence supporting the use of SGLT2 inhibitors in HF management. In the DAPA-HF trial, dapagliflozin reduced the composite outcome of cardiovascular death or worsening heart failure by 26% (HR 0.74; 95% CI 0.65–0.85) compared with placebo, with a number needed to treat (NNT) of approximately 21 over 18 months (McMurray et al., 2019). Similarly, the EMPEROR-Reduced trial demonstrated that empagliflozin reduced the same composite endpoint by 25% (HR 0.75; 95% CI 0.65–0.86) (Packer et al., 2021). Importantly, these benefits were observed regardless of the presence of diabetes, supporting the repositioning of SGLT2 inhibitors as cardiovascular therapies rather than solely glucose-lowering agents. Subsequent evidence has extended these benefits to patients with heart failure with preserved ejection fraction (HFpEF), positioning SGLT2 inhibitors as a foundational therapy across the HF spectrum (Minisy and Abdelaziz, 2025).

Reflecting this strong evidence base, recent international guidelines now recommend SGLT2 inhibitors as a foundational therapy for HFrEF. Both the 2022 American College of Cardiology/American Heart Association (ACC/AHA) guidelines and the 2023 European Society of Cardiology (ESC) focused updates identify four key pillars of guideline-directed medical therapy: (1) renin–angiotensin system inhibition with ACE inhibitors, ARNI, or ARBs, (2) beta-blockers, (3) mineralocorticoid receptor antagonists, and (4) SGLT2 inhibitors. These therapies are recommended for early and simultaneous initiation when clinically appropriate due to their complementary mechanisms and additive benefits in reducing mortality and heart failure hospitalizations (Ponikowski et al., 2016; McDonald et al., 2021).

Despite the strength and consistency of this evidence, real-world uptake of SGLT2 inhibitors for HF remains suboptimal (Greene et al., 2018). In Jordan, prior research has shown that SGLT2 inhibitors are the least commonly prescribed component of the four pillars of guideline-directed heart failure therapy (Izraiq et al., 2024), with SGLT2 inhibitors remaining the least commonly prescribed component of guideline-directed therapy, utilized in only 9% of HF patients in the Jordan Heart Failure Registry ^3^. Previous studies have shown that barriers to implementation extend beyond clinical considerations and include physician-related factors (knowledge, confidence, role perception), system-level constraints (drug availability, reimbursement policies), and administrative processes that influence prescribing and dispensing decisions (Dhaliwal et al., 2022; Yi et al., 2024; Greene and Pierce, 2023; Milder et al., 2022; Teng et al., 2024). Importantly, much of the existing literature has focused on cardiologists’ prescribing behavior, while relatively little attention has been paid to general physicians, family physicians, and internist clinicians who often represent the first point of contact for patients with HF and play a central role in initiating and coordinating long-term pharmacotherapy.

In many healthcare systems, including Jordan, prescribing decisions do not occur in isolation. Authorization and reimbursement processes, particularly for high-cost medications, introduce an additional layer of clinical decision-making that may influence whether evidence-based therapies ultimately reach patients. In this context, medical officers working within health insurance companies act as gatekeepers, evaluating prescriptions and determining coverage approval. Anecdotal clinical experience in Jordan suggests that SGLT2 inhibitors prescribed for HF are frequently perceived as “diabetes-only medications” during authorization reviews, leading to rejection of coverage despite guideline-supported indications. Such misalignment between contemporary evidence and authorization practices has the potential to undermine optimal HF management, delay initiation of life-saving therapies, and exacerbate inequities in care.

To date, no study in Jordan, and very few in the Middle East and North Africa region, has systematically explored the perspectives of both treating physicians and insurance-based medical reviewers regarding the use of SGLT2 inhibitors for HF. Understanding how knowledge, attitudes, prescribing practices, and authorization criteria intersect across these stakeholder groups is essential for identifying system-level barriers to evidence-based pharmacotherapy. Moreover, integrating quantitative assessment with qualitative inquiry may provide deeper insight into the underlying reasons for variability in prescribing and approval decisions, beyond what can be captured through surveys alone.

Therefore, this study aimed to explore the knowledge, attitudes, and prescribing practices of treating physicians toward SGLT2 inhibitors for heart failure in Jordan, alongside the perspectives and authorization decision-making processes of insurance physicians. Using a mixed-methods approach, we sought to identify clinical, regulatory, and insurance-related factors associated with initiation or rejection of SGLT2 inhibitors for HF, and to examine the degree of alignment between real-world practice and contemporary guideline recommendations. By addressing this underexplored interface between evidence, prescribing, and authorization, this study aims to inform targeted interventions and policy-level strategies to optimize heart failure pharmacotherapy in Jordan and similar healthcare settings.

## 2 Methods

### 2.1 Study design

This study adopted an explanatory sequential mixed-methods design, in which a quantitative cross-sectional survey was conducted first, followed by a qualitative phase using semi-structured interviews. This design was selected to enable systematic exploration of physicians’ knowledge, attitudes, prescribing behaviors, and authorization practices regarding SGLT2 inhibitors in HF, and to subsequently explain observed quantitative patterns through in-depth qualitative inquiry. Integration of quantitative and qualitative findings occurred at the interpretation stage to provide a comprehensive understanding of the evidence–practice gap in the Jordanian healthcare context.

### 2.2 Study setting and participants

The study was conducted in Jordan between June and November 2025. Two physician groups were targeted because of their complementary roles in determining access to HF pharmacotherapy. The first group comprised treating physicians, including general practitioners, family medicine physicians, and internal medicine physicians involved in direct care of adult patients with heart failure. The second group comprised insurance physicians employed by health insurance providers or third-party administrators who were responsible for reviewing and approving medication reimbursement requests. Authorization workflows in Jordan often involve multidisciplinary personnel, including nursing and pharmacy staff who participate in preliminary review stages. However, complex or disputed cases are escalated to a designated medical director, and final authorization decisions are formally attributed to a physician. Because authorization decisions are framed as medical determinations regarding prescription legitimacy and indication appropriateness, the present study focused on physicians as the formal locus of clinical accountability within authorization systems. Physicians who did not participate in prescribing decisions related to cardiovascular medications were excluded. Participation was voluntary, and no identifying information regarding participants or institutions was collected. Participants were recruited using a convenience sampling approach through professional networks, clinical contacts, and direct outreach within the Jordanian healthcare system (including hospitals, clinics, and insurance providers). Potential participants were identified and invited via email, WhatsApp professional groups, and in-person announcements at relevant medical meetings and continuing medical education events. No incentives were offered, and participation was entirely voluntary. Participation was voluntary, and no identifying information regarding participants or institutions was collected.

### 2.3 Sample size calculation

Given the exploratory mixed-methods design, no formal *a priori* sample size calculation was performed to detect small between-group differences. However, prior to analysis, the minimum sample required to detect a moderate difference in authorization outcomes between groups was estimated. Assuming a baseline approval rate of approximately 35%, a clinically meaningful absolute difference of 15%, a two-sided α of 0.05, and 80% power, at least 90 participants per group were required (using standard two-proportion sample size formulas). The final sample of 312 physicians (214 treating physicians and 98 insurance physicians) met this requirement and was sufficient for planned inferential analyses and multivariable logistic regression, with acceptable events-per-variable ratios (>10) for all models.

### 2.4 Quantitative instrument development

The questionnaire was developed using a theoretically informed approach grounded primarily in the Capability–Opportunity–Motivation–Behavior (COM-B) framework to capture cognitive, motivational, behavioral, and contextual determinants of prescribing and authorization practices. The Knowledge–Attitude–Practice (KAP) structure additionally informed the organizational flow of the instrument but did not serve as the primary theoretical model. The questionnaire was developed by the research team with expertise in clinical pharmacy, cardiology pharmacotherapy, and health services research. The team included academic clinical pharmacists with experience in cardiovascular pharmacotherapy research as well as investigators with expertise in survey methodology and mixed-methods research.

Item development was informed by contemporary international HF guidelines, prior studies examining uptake of SGLT2 inhibitors, and real-world prescribing and authorization workflows within the Jordanian healthcare system (Ponikowski et al., 2016; McDonald et al., 2021; Greene and Pierce, 2023; Teng et al., 2024; Pradhan et al., 2024). An initial pool of 52 items was generated through an extensive literature review and iterative discussion among the research team. Items were designed to capture six core domains including physician and practice characteristics, knowledge of SGLT2 inhibitors in HF, attitudes toward prescribing and approval, real-world prescribing and authorization practices, insurance-related barriers, and perceptions of drug misclassification.

Knowledge items were constructed as binary (true/false) or single-best-answer questions to facilitate objective scoring (maximum score = 10). Attitudinal and barrier-related items were measured using five-point Likert scales (1 = strongly disagree to 5 = strongly agree) to capture perceptions related to professional role identity, prescribing confidence, risk–benefit evaluation, institutional influences, and reimbursement dynamics. Practice-related items employed frequency scales, categorical decision pathways, and likelihood ratings to reflect real-world clinical and administrative behaviors. All items were worded to ensure applicability to both treating physicians and insurance physicians, with role-specific adaptations implemented where appropriate.

Content validity was established through structured expert review by an independent panel (two cardiologists, one clinical pharmacist specializing in heart failure pharmacotherapy, one academic pharmacologist, and one physician with direct insurance authorization experience). Each item was rated for relevance, clarity, and contextual appropriateness on a 4-point scale. Item-level Content Validity Index (CVI) and scale-level CVI were calculated; items with item-level CVI <0.78 were revised or removed. Following feedback, nine items were eliminated due to redundancy or limited relevance, and six were reworded for improved clarity and alignment with the Jordanian context. The finalized questionnaire comprised 46 items. The full questionnaire is provided in Supplementary Appendix I.

The revised questionnaire was pilot-tested with 20 physicians who met eligibility criteria but were not included in the final analysis. Minor linguistic and formatting modifications were implemented based on participant feedback. Internal consistency reliability was assessed using Cronbach’s alpha coefficients. The knowledge scale demonstrated good reliability (α = 0.81), the attitude scale showed strong internal consistency (α = 0.84), and the insurance-related barrier scale demonstrated acceptable reliability (α = 0.79).

### 2.5 Quantitative data collection and analysis

The final questionnaire was administered electronically using a secure online platform. Data were analyzed using SPSS version 26. Descriptive statistics summarized participant characteristics and response distributions. Group comparisons were conducted using chi-square or Fisher’s exact tests for categorical variables and independent t-tests or Mann–Whitney U tests for continuous variables, as appropriate. Composite knowledge and attitude scores were calculated by summing relevant items. Multivariable logistic regression analyses were performed to identify independent predictors of SGLT2 inhibitor initiation by treating physicians and approval of SGLT2 inhibitor prescriptions by insurance providers. Variables were entered into regression models based on theoretical relevance and univariable associations (p < 0.20). Adjusted odds ratios with 95% confidence intervals were reported.

### 2.6 Qualitative phase and interview guide development

Following preliminary quantitative analysis, a purposive sampling strategy was used to select participants for the qualitative phase. Selection was informed by survey responses to ensure variation in knowledge levels, attitudes, prescribing behavior, authorization roles, practice settings, and approval tendencies. This approach ensured that qualitative data would directly address areas of divergence and uncertainty identified in the quantitative results.

The semi-structured interview guide was developed iteratively based on key quantitative findings, existing theoretical constructs related to clinical decision-making and risk perception, and pilot discussions within the research team. The guide explored perceptions of SGLT2 inhibitors in HF, role boundaries in prescribing and authorization, interpretation of guidelines, experiences with rejection and appeals, and views on system-level facilitators and barriers. Open-ended questions were used throughout, with probes applied flexibly to explore emerging issues in depth.

### 2.7 Qualitative data collection, translation, and transcription

A total of 22 interviews were conducted, including 12 treating physicians and 10 insurance physicians. Interviews were conducted in person, lasted approximately 30–45 min, and were audio-recorded with participant consent. Interviews were conducted in Arabic. All recordings were transcribed verbatim, and transcripts were translated into English by a bilingual researcher with clinical and methodological expertise. To ensure translation accuracy and conceptual equivalence, translated transcripts were independently reviewed against the original audio by a second bilingual researcher. Discrepancies were discussed and resolved through consensus, with attention to preserving contextual meaning rather than literal wording.

### 2.8 Qualitative coding and analysis

Qualitative data were analyzed using reflexive thematic analysis following the framework proposed by Braun and Clarke. Analysis began with repeated reading of transcripts to achieve familiarization. Initial coding was conducted inductively, allowing codes to emerge from the data rather than being imposed *a priori*. Coding was performed independently by two researchers using NVivo version 12. After initial coding, the research team met to compare code lists, resolve discrepancies, and refine code definitions. Codes were then grouped into candidate themes, which were iteratively reviewed against the data set to ensure coherence and distinctiveness. Themes were refined through ongoing discussion and reflexive consideration of alternative interpretations until consensus was achieved. An audit trail documenting coding decisions, theme development, and analytic memos was maintained throughout the process. Data saturation was assessed continuously and was reached after 20 interviews, with no substantively new themes emerging in subsequent interviews.

### 2.9 Ethical considerations

This study was performed in line with the principles of the Declaration of Helsinki. Approval was granted by the Ethics Committee of Al-Ahliyya Amman University (IRB No. AAU/1/8/2024-2025). Participation was voluntary, informed consent was obtained prior to data collection, and confidentiality was strictly maintained throughout the study.

## 3 Results

### 3.1 Characteristics of the study population and practice settings

A total of 312 physicians participated in the quantitative component of the study, comprising 214 treating physicians and 98 insurance physicians. As shown in Table 1, among treating physicians, general practitioners (GPs) constituted 46.7%, followed by family medicine physicians (31.3%) and internal medicine physicians (22.0%). Treating physicians were nearly evenly split between public-sector practice (51.4%) and private-sector practice (48.6%). In contrast, insurance physicians were exclusively employed within private insurance organizations and reported no concurrent clinical duties during authorization shifts.

**TABLE 1:** Characteristics of the study population and practice settings.

Characteristic	Treating physicians (n = 214)	Insurance physicians (n = 98)	p-value
Age, years (mean ± SD)	36.8 ± 8.4	35.2 ± 7.9	0.18
Years of professional experience, median (IQR)	8 (4–14)	6 (3–11)	0.04
Primary specialty, n (%)	​	​	<0.001
– General practice	100 (46.7)	98 (100.0)	​
– Family medicine	67 (31.3)	​	​
– Internal medicine	47 (22.0)	​	​
Primary work sector, n (%)	​	​	<0.001
– Public (MoH/RMS/University hospitals)	110 (51.4)	0 (0.0)	​
– Private	104 (48.6)	98 (100.0)	​
Direct clinical care of HF patients, n (%)	214 (100.0)	0 (0.0)	<0.001
Number of HF patients/month, median (IQR)	18 (10–30)	Not applicable	—
Formal HF-related CME in past 3 years, n (%)	83 (38.8)	21 (21.4)	0.002
Routine use of international HF guidelines, n (%)^a^	94 (43.9)	23 (23.5)	<0.001
Authority to prescribe SGLT2 inhibitors, n (%)	156 (72.9)	Not applicable	—
Involvement in authorization decisions for high-cost drugs, n (%)	41 (19.2)	98 (100.0)	<0.001
Perceived autonomy in initiating HF pharmacotherapy, n (%)	​	​	<0.001
– High	68 (31.8)	0 (0.0)	​
– Moderate	97 (45.3)	14 (14.3)	​
– Low	49 (22.9)	84 (85.7)	​
Prescriber specialty influences approval decisions^b^, n (%)	138 (64.5)	63 (64.3)	0.97
SGLT2 inhibitors usually approved for HF without diabetes, n (%)	89 (41.6)	37 (37.8)	0.56

^a^ International HF, guidelines include ESC, and ACC/AHA, recommendations.

^b^ Refers to the perception (treating physicians) or reported practice (insurance physicians) that cardiologist-issued prescriptions are more likely to be approved.

The median duration of clinical experience among treating physicians was 8 years, compared with 6 years among insurance physicians. Despite similar years since graduation, marked differences were observed in clinical exposure to HF. Treating physicians reported a median of 18 HF patients per month, whereas insurance physicians reported indirect exposure limited to chart review and authorization requests, without direct patient contact.

Only 38.8% of treating physicians reported having received formal training or continuing medical education (CME) related to contemporary HF pharmacotherapy within the past 3 years. This proportion was significantly lower among insurance physicians (21.4%), the majority of whom indicated that their decision-making relied primarily on internal insurance policies, drug lists, or predefined indication codes, rather than updated clinical guidelines. Notably, fewer than one-quarter (23.5%) of insurance physicians reported routinely consulting international HF guidelines when reviewing authorization requests.

Practice setting exerted a clear influence on clinical responsibility and autonomy. Treating physicians working in governmental hospitals or primary healthcare centers frequently described restricted prescribing authority, particularly for high-cost medications requiring prior authorization. Conversely, private-sector physicians reported greater autonomy in initiating therapy but acknowledged that reimbursement decisions remained outside their control. This structural separation between prescribing and approval roles created a recurring interface where treatment decisions were subject to secondary clinical interpretation by insurance physicians.

Importantly, although most treating physicians reported that they were legally permitted to prescribe SGLT2 inhibitors within their institutions, only 41.6% indicated that such prescriptions were usually approved by insurance providers when written for heart failure indications without concomitant diabetes mellitus. Treating physicians frequently described automatic rejection patterns linked to diagnosis–drug mismatches within digital authorization systems, requiring additional justification or negotiation for approval. Among insurance physicians, 64.3% reported that authorization decisions were operationally influenced by the prescriber’s specialty, with cardiologist-issued prescriptions more likely to receive approval than those initiated by general or family physicians, despite the absence of formal regulatory restrictions on prescribing authority.

### 3.2 Knowledge of SGLT2 inhibitors for heart failure among treating and insurance physicians

Overall knowledge regarding the role of SGLT2 inhibitors in heart failure varied substantially between treating physicians and insurance physicians. As elucidate in Table 2, treating physicians achieved a significantly higher mean score compared with insurance physicians (6.7 ± 1.8 vs. 4.9 ± 1.9, p < 0.001). However, even among treating physicians, knowledge gaps were evident in several clinically critical domains.

**TABLE 2:** Knowledge of SGLT2 inhibitors for heart failure among treating physicians and insurance physicians.

Knowledge item	Treating physicians (n = 214)	Insurance physicians (n = 98)	p-value
Overall knowledge score (0–10), mean ± SD	6.7 ± 1.8	4.9 ± 1.9	<0.001
Aware that SGLT2 inhibitors reduce HF hospitalization independent of diabetes, n (%)	153 (71.5)	38 (38.8)	<0.001
Recognize SGLT2 inhibitors as guideline-recommended for HFrEF, n (%)	137 (64.0)	34 (34.7)	<0.001
Recognize SGLT2 inhibitors as beneficial in HFpEF, n (%)	99 (46.3)	19 (19.4)	<0.001
Correctly identify SGLT2 inhibitors as not limited to diabetes-only indication, n (%)	165 (77.1)	38 (38.8)	<0.001
Misclassify SGLT2 inhibitors as “diabetes-only drugs”, n (%)	49 (22.9)	60 (61.2)	<0.001
Correctly identify eGFR threshold for initiation in HF, n (%)	125 (58.4)	27 (27.6)	<0.001
Incorrectly believe SGLT2 inhibitors are contraindicated in all CKD patients, n (%)	41 (19.2)	41 (41.8)	<0.001
Aware of ESC/ACC HF guideline updates within last 3 years, n (%)	94 (43.9)	23 (23.5)	<0.001
Higher knowledge score among those with recent HF-related CME, mean ± SD^a^	7.4 ± 1.5	5.8 ± 1.6	—

^a^ HF-related CME, refers to formal educational activities addressing contemporary heart failure pharmacotherapy within the past 3 years.

Awareness that SGLT2 inhibitors reduce HF hospitalizations independent of diabetes status was reported by 71.5% of treating physicians, compared with only 38.8% of insurance physicians (p < 0.001). Similarly, recognition of SGLT2 inhibitors as guideline-recommended therapy for HFrEF was significantly higher among treating physicians (64.0%) than among insurance physicians (34.7%, p < 0.001). Knowledge deficits were more pronounced for HFpEF, with fewer than half of treating physicians (46.3%) and only 19.4% of insurance physicians correctly identifying SGLT2 inhibitors as evidence-supported therapy for this phenotype.

Misclassification of SGLT2 inhibitors as “diabetes-only medications” emerged as a prominent knowledge gap. While 22.9% of treating physicians endorsed this misconception, the proportion was markedly higher among insurance physicians (61.2%, p < 0.001). This misperception was strongly associated with lower overall knowledge scores and was later shown to correlate with higher rates of authorization rejection.

Knowledge related to renal function thresholds further illustrated discordance between groups. Correct identification of the estimated glomerular filtration rate (eGFR) threshold permitting safe initiation of SGLT2 inhibitors in HF was reported by 58.4% of treating physicians, compared with 27.6% of insurance physicians (p < 0.001). Notably, 41.8% of insurance physicians incorrectly believed that SGLT2 inhibitors were contraindicated in all patients with chronic kidney disease, reflecting reliance on outdated prescribing paradigms.

Stratified analysis among treating physicians demonstrated that higher knowledge scores were significantly associated with prior HF-related CME (p < 0.001) and routine consultation of international HF guidelines (p < 0.001). In contrast, among insurance physicians, years of professional experience were not associated with higher knowledge scores, whereas participation in formal guideline-oriented training showed a modest but significant association with improved knowledge (p = 0.03).

### 3.3 Attitudes toward the use of SGLT2 inhibitors in heart failure

Attitudinal assessment revealed marked differences between treating physicians and insurance physicians regarding confidence, perceived responsibility, and risk associated with the use of SGLT2 inhibitors in HF (Table 3). Treating physicians demonstrated significantly more positive attitudes compared with insurance physicians (p < 0.001). Among treating physicians, 61.2% agreed or strongly agreed that SGLT2 inhibitors should be initiated early as part of guideline-directed medical therapy for HF. However, only 34.7% reported feeling fully confident initiating SGLT2 inhibitors without prior cardiology consultation. This ambivalence was particularly pronounced in public-sector physicians, where concerns regarding authorization rejection and medico-legal accountability were frequently cited. In contrast, insurance physicians expressed significantly lower confidence in approving SGLT2 inhibitors for HF indications. Only 28.6% agreed that SGLT2 inhibitors represent standard therapy for HF irrespective of diabetes status, while 57.1% indicated that they perceived such prescriptions as falling outside the “core indication” of the drug class. This perception was strongly aligned with the previously identified knowledge gap and was independent of years of professional experience.

**TABLE 3:** Attitudes toward the use of SGLT2 inhibitors for heart failur**e**.

Attitudinal item	Treating physicians (n = 214)	Insurance physicians (n = 98)	p-value
Overall attitude score (1–5), mean ± SD	3.6 ± 0.7	2.8 ± 0.8	<0.001
Agree that SGLT2 inhibitors should be initiated early in HF, n (%)	131 (61.2)	28 (28.6)	<0.001
Feel confident initiating/approving SGLT2 inhibitors for HF without cardiology input, n (%)	74 (34.7)	19 (19.4)	0.006
Believe SGLT2 inhibitors are standard HF therapy irrespective of diabetes, n (%)	149 (69.6)	28 (28.6)	<0.001
Perceive SGLT2 inhibitors for HF as outside the drug’s “core indication”, n (%)	55 (25.7)	56 (57.1)	<0.001
Agree that initiation should be restricted to cardiologists, n (%)	63 (29.4)	62 (63.3)	<0.001
Reluctant to initiate due to fear of authorization rejection, n (%)	88 (41.1)	Not applicable	—
Concerned that risks outweigh benefits in non-diabetic HF, n (%)	51 (23.8)	53 (54.1)	<0.001
Attitudes influenced primarily by internal policies/cost considerations, n (%)	47 (22.0)	71 (72.4)	<0.001

Perceived role boundaries emerged as a central attitudinal theme. A majority of insurance physicians (63.3%) agreed that initiation of SGLT2 inhibitors for HF should be restricted to cardiologists, compared with 29.4% of treating physicians (p < 0.001). Notably, even among treating physicians, 41.1% reported reluctance to initiate SGLT2 inhibitors due to concerns that doing so would be viewed as exceeding their professional remit, particularly in the absence of explicit institutional protocols.

Risk aversion further differentiated attitudes between groups. Concerns regarding adverse effects—such as hypotension, acute kidney injury, and diabetic ketoacidosis—were reported by both groups but were significantly more prevalent among insurance physicians. Over half of insurance physicians (54.1%) agreed that potential risks outweighed benefits in non-diabetic HF patients, compared with 23.8% of treating physicians (p < 0.001). Importantly, these concerns persisted despite acknowledgment of guideline recommendations among a subset of respondents, suggesting that attitudes were shaped not only by knowledge but also by institutional and economic considerations.

Stratified analysis demonstrated that favorable attitudes among treating physicians were strongly associated with higher knowledge scores (Spearman’s ρ = 0.48, p < 0.001) and prior HF-related CME (p = 0.002). Among insurance physicians, however, attitudes were more closely associated with internal policy frameworks and perceived cost implications than with clinical training or guideline familiarity.

### 3.4 Prescribing and authorization practices in real-world heart failure care

Marked discrepancies were observed between intended prescribing practices among treating physicians and actual authorization outcomes for SGLT2 inhibitors in HF (Table 4). Among treating physicians, 57.0% reported having prescribed or attempted to prescribe an SGLT2 inhibitor for HF at least once during the preceding 12 months. However, only 32.7% indicated that these prescriptions were “consistently approved” by insurance providers when the indication was HF without concomitant diabetes mellitus.

**TABLE 4:** Prescribing and authorization practices for SGLT2 inhibitors in heart failur**e**.

Practice characteristic	Treating physicians (n = 214)	Insurance physicians (n = 98)	p-value
Prescribed/attempted to prescribe SGLT2 inhibitors for HF in past 12 months, n (%)	122 (57.0)	Not applicable	—
Prescriptions consistently approved for HF without diabetes, n (%)	70 (32.7)	37 (37.8)	0.43
Initiate SGLT2 inhibitors independently without cardiology referral, n (%)	71 (33.2)	Not applicable	—
Defers initiation pending cardiology consultation, n (%)	95 (44.4)	Not applicable	—
Initiation differs by sector (private vs. public), n (%)	48.9 vs. 21.6	—	<0.001
Authorization approval rate by prescriber specialty, %	​	​	<0.001
– Cardiologist	71.4	73.5	​
– Internal medicine	38.2	36.7	​
– GP/Family medicine	29.6	28.6	​
Require cardiologist documentation for approval, n (%)	—	67 (68.4)	—
Approval less likely in absence of diabetes diagnosis, n (%)	—	60 (61.2)	—
Presence of standardized internal protocol recognizing HF indication, n (%)	—	26 (26.5)	—
Common reasons for rejection (multiple responses), n (%)			
– Indication not aligned with drug class	71 (58.2)	57 (58.2)	—
– Prescriber specialty not appropriate	57 (46.9)	46 (46.9)	—
– Cost-containment considerations	52 (42.9)	49 (50.0)	—
– Clinical safety concerns	29 (23.8)	22 (22.4)	—

Initiation practices varied substantially by practice setting. Treating physicians in the private sector were significantly more likely to initiate SGLT2 inhibitors independently compared with those in public-sector settings (p < 0.001). Public-sector physicians more frequently reported deferring initiation until cardiology consultation (p < 0.001), citing anticipated authorization rejection and institutional prescribing restrictions as primary reasons for delay.

Authorization outcomes demonstrated a clear dependence on prescriber specialty. Prescriptions initiated by cardiologists were reported as approved in 71.4% of cases, compared with 38.2% for internal medicine physicians and 29.6% for general or family physicians (p < 0.001). This pattern persisted after stratification by sector and years of experience, suggesting that specialty-based authorization bias represented a systemic rather than individual phenomenon.

From the perspective of insurance physicians, 68.4% reported that they “frequently” or “almost always” required documentation from a cardiologist to approve SGLT2 inhibitors for HF. Furthermore, 61.2% indicated that the absence of a diabetes diagnosis significantly reduced the likelihood of approval, even when HF documentation and echocardiographic data were provided. Notably, only 26.5% of insurance physicians reported having a standardized internal protocol explicitly recognizing HF as an approved indication for SGLT2 inhibitors.

Rejection patterns revealed that insurance-related factors outweighed clinical considerations. The most commonly cited reasons for rejection included “indication not aligned with drug class” (58.2%), “prescriber specialty not appropriate” (46.9%), and “cost-containment policies” (42.9%). In contrast, purely clinical concerns—such as renal function or blood pressure—were cited in fewer than one-quarter of rejection decisions.

Taken together, these findings illustrate a substantial implementation gap between guideline-supported prescribing intent and real-world access to therapy. While treating physicians increasingly recognize the role of SGLT2 inhibitors in HF, authorization practices, shaped by specialty hierarchies, diagnostic framing, and insurance policy constraints, emerge as dominant determinants of whether treatment is ultimately initiated. This gap underscores the need to examine not only prescribing behavior but also the administrative processes that mediate access to evidence-based pharmacotherapy.

### 3.5 Misclassification of SGLT2 inhibitors as diabetes-only medications during authorization

Misclassification of SGLT2 inhibitors as medications indicated exclusively for diabetes mellitus emerged as a central and recurrent barrier influencing authorization decisions for HF. This phenomenon was consistently reported by both treating physicians and insurance physicians, though with markedly different prevalence and implications across groups.

As shown in Table 5, among insurance physicians, 61.2% explicitly classified SGLT2 inhibitors as “diabetes-only medications,” despite the presence of HF-specific documentation in authorization requests. In comparison, 22.9% of treating physicians endorsed the same classification (p < 0.001). Importantly, this misclassification was not merely conceptual but translated directly into authorization outcomes. Prescriptions classified as diabetes-only were associated with a substantially higher rejection rate compared with those in which the heart failure indication was recognized (74.6% vs. 28.1%, p < 0.001), indicating that diagnostic misclassification was the primary driver of rejection rather than physician role. However, rejection rates did not differ significantly between treating physicians and insurance physicians when prescriptions were classified as diabetes-only (74.6% vs. 72.1%, p = 0.81) or when the heart failure indication was recognized (28.1% vs. 31.4%, p = 0.67).

**TABLE 5:** Misclassification of SGLT2 inhibitors as diabetes-only medications and its impact on authorizatio**n**.

Misclassification-related indicator	Treating physicians (n = 214)	Insurance physicians (n = 98)	p-value
Classify SGLT2 inhibitors as “diabetes-only medications”, n (%)	49 (22.9)	60 (61.2)	<0.001
Authorization rejection when classified as diabetes-only, %	74.6	72.1	0.81
Authorization rejection when HF indication recognized, %	28.1	31.4	0.67
Misclassification among officers relying on internal drug lists, n (%)	—	40 (78.4)	—
Misclassification among officers consulting HF guidelines, n (%)	—	10 (33.3)	<0.001
Absence of diabetes diagnosis reduces approval likelihood, n (%)	125 (58.4)	57 (58.2)	0.97
Repeated rejection discourages future prescribing attempts, n (%)^a^	83 (38.8)	Not applicable	—
SGLT2 inhibitors viewed as off-label for HF, n (%)	41 (19.2)	46 (46.9)	<0.001
Belief that cardiologist endorsement overrides classification, n (%)	146 (68.2)	71 (72.4)	0.47

^a^ Refers to treating physicians reporting reduced likelihood of re-attempting SGLT2 inhibitors initiation for HF, following prior authorization rejection attributed to diabetes-only classification.

Further analysis revealed that misclassification was strongly associated with reliance on drug class labelling rather than guideline-based indication assessment. Among insurance physicians who reported primary dependence on internal drug lists or indication codes, 78.4% misclassified SGLT2 inhibitors, compared with 33.3% among those who reported routine consultation of international HF guidelines (p < 0.001). This finding suggests that misclassification reflects systemic decision frameworks rather than isolated knowledge deficits.

The clinical consequences of misclassification were evident in prescribing behavior. Treating physicians who reported repeated authorization rejections attributed to diabetes-only classification were significantly less likely to attempt re-initiation of SGLT2 inhibitors for HF (21.6% vs. 49.2%, p < 0.001), indicating a deterrent effect that extended beyond individual cases. Qualitative comments embedded within survey responses further suggested that repeated rejection fostered therapeutic inertia and reinforced perceptions that prescribing SGLT2 inhibitors for HF was “futile” outside cardiology-led pathways. This concern is particularly relevant in the Jordanian context, where effective heart failure management relies heavily on continuity of care and patient self-management, both of which are sensitive to delays and disruptions in access to guideline-directed therapies (Alkouri et al., 2022).

Notably, misclassification persisted even in cases where HF was clearly documented as the primary indication. Among insurance physicians, 58.2% reported that absence of a diabetes diagnosis substantially reduced approval likelihood, even when echocardiographic data and cardiology consultation notes were provided. This pattern highlights a discordance between contemporary evidence and authorization logic, wherein indication assessment is anchored to historical drug classification rather than evolving pharmacotherapeutic roles.

Collectively, these findings identify misclassification of SGLT2 inhibitors as diabetes-only medications as a dominant mechanism through which evidence-based HF therapy is obstructed at the authorization level. This mechanism operates independently of clinical complexity and disproportionately affects prescriptions initiated by non-cardiologist physicians, thereby amplifying inequities in access to guideline-directed medical therapy.

### 3.6 Insurance-related barriers to evidence-based use of SGLT2 inhibitors in heart failure

As shown in Table 6, insurance-related constraints emerged as dominant system-level barriers influencing access to SGLT2 inhibitors for HF, superseding clinical considerations in a substantial proportion of authorization decisions. Both treating physicians and insurance physicians consistently identified prior authorization requirements, indication coding practices, and cost-containment policies as key determinants of approval outcomes.

**TABLE 6:** Insurance-related barriers to the use of SGLT2 inhibitors for heart failur**e**.

Barrier or policy characteristic	Treating physicians (n = 214)	Insurance physicians (n = 98)	p-value
Prior authorization perceived as major barrier, n (%)	145 (67.8)	—	—
Treatment initiation delayed >4 weeks due to authorization, n (%)	127 (59.3)	—	—
Cost considerations substantially influence approval decisions, n (%)	92 (43.0)	71 (72.4)	<0.001
HF without diabetes viewed as lower-priority indication, n (%)	118 (55.1)	63 (64.3)	0.14
Presence of internal protocol recognizing HF indication, n (%)	—	26 (26.5)	—
Reliance on ICD coding over clinical documentation, n (%)	101 (47.2)	58 (59.2)	0.06
Require echocardiography report for approval, n (%)	152 (71.0)	69 (70.4)	0.92
Require cardiology consultation note, n (%)	146 (68.4)	67 (68.4)	1.00
Extensive documentation beyond routine care required, n (%)	112 (52.3)	49 (50.0)	0.72
Formal appeals process available, n (%)	—	34 (34.7)	—
Appeals frequently lead to approval reversal, n (%)^a^	—	14 (41.2)	—
Conditional or time-limited approvals common, n (%)	100 (46.7)	45 (45.9)	0.90

^a^ Percentage calculated among insurance physicians reporting the existence of a formal appeals process.

Among treating physicians, 67.8% reported that prior authorization requirements constituted a “major barrier” to initiating SGLT2 inhibitors for HF, while 59.3% indicated that repeated rejection led to delays in treatment initiation exceeding 4 weeks. These delays were most frequently reported in public-sector settings, where administrative pathways were described as more rigid and less responsive to clinical justification.

From the perspective of insurance physicians, 72.4% reported that cost considerations played a “substantial” or “very substantial” role in authorization decisions for SGLT2 inhibitors. Importantly, 64.3% acknowledged that cost-related pressures occasionally overrode clinical arguments, particularly when the indication was HF without diabetes mellitus. Only 29.6% reported that their organizations maintained explicit policies aligning SGLT2 inhibitor approval with contemporary HF guidelines.

The absence of standardized authorization protocols was a recurring theme. Less than one-third of insurance physicians (26.5%) indicated the presence of a written internal protocol recognizing HF as a primary indication for SGLT2 inhibitors. In the absence of such protocols, decisions were frequently based on ICD coding, prescriber specialty, or historical drug classification, reinforcing the misclassification patterns identified in Result 5.

Documentation burden further amplified access barriers. Treating physicians reported that approval typically required multiple supporting documents, including echocardiography reports (71.0%), cardiology consultation notes (68.4%), and laboratory results exceeding routine clinical requirements (52.3%). Despite comprehensive documentation, 46.7% of treating physicians reported persistent rejection or conditional approval (e.g., limited duration coverage), suggesting that administrative completeness alone was insufficient to secure access.

Notably, there was limited evidence of structured appeal mechanisms. Only 34.7% of insurance physicians reported the existence of a formal appeals process for rejected HF prescriptions, and among those, 41.2% indicated that appeals rarely resulted in approval reversal. This lack of effective recourse contributed to therapeutic inertia, as treating physicians increasingly opted to defer initiation or avoid prescribing SGLT2 inhibitors altogether.

### 3.7 Factors associated with initiation and approval of SGLT2 inhibitors for heart failure

Multivariable logistic regression analysis was performed to identify independent factors associated with initiation of SGLT2 inhibitors by treating physicians and approval of SGLT2 inhibitors prescriptions by insurance providers for HF. Variables entered into the model were selected *a priori* based on clinical relevance and findings from univariable analyses, including physician role, knowledge and attitude scores, prior HF-related training, practice sector, perceived misclassification, and insurance-related policy factors.

As shown in Table 7, higher knowledge scores were independently associated with increased likelihood of initiating SGLT2 inhibitors for HF. Favorable attitudes toward SGLT2 inhibitors were similarly associated with initiation. Treating physicians who had received HF-related CME within the preceding 3 years were significantly more likely to initiate therapy. In contrast, perceived likelihood of insurance rejection emerged as a strong negative factor associated with initiation, outweighing the influence of both knowledge and training. Practice sector also played a role, with public-sector physicians less likely to initiate SGLT2 inhibitors compared with their private-sector counterparts.

**TABLE 7:** Multivariable logistic regression analysis of factors associated with initiation and approval of SGLT2 inhibitors for heart failur**e**.

Predictor	aOR	95% CI	p-value
Factors associated with initiation by treating physicians (n = 214)			
Predictor	aOR	95% CI	p-value
Knowledge score (per 1-point increase)	1.42	1.19–1.69	<0.001
Attitude score (per 1-point increase)	1.36	1.11–1.67	0.003
HF-related CME (yes vs. no)	1.89	1.12–3.18	0.017
Public-sector practice (vs. private)	0.58	0.36–0.93	0.024
High perceived likelihood of rejection	0.41	0.26–0.64	<0.001
Years of experience	1.02	0.98–1.06	0.32
Factors associated with authorization approval (n = 159 prescriptions)			
Misclassification as diabetes-only drug	0.18	0.10–0.33	<0.001
Absence of internal HF approval protocol	0.31	0.17–0.58	<0.001
Non-cardiologist prescriber	0.44	0.26–0.75	0.003
Complete HF documentation provided	1.21	0.72–2.03	0.47
Cost flagged as primary concern	0.62	0.38–1.01	0.056
Insurance officer guideline familiarity	1.34	0.81–2.22	0.25

Among prescriptions submitted for authorization, misclassification of SGLT2 inhibitors as diabetes-only medications was the factor most strongly associated with rejection. Prescriptions subject to misclassification had an 82% lower likelihood of approval, independent of prescriber specialty, documentation completeness, or clinical indication.

Absence of a standardized internal insurance protocol recognizing HF as an approved indication was also independently associated with rejection. Prescriptions initiated by non-cardiologist physicians were less likely to be approved, even after adjustment for patient documentation and disease severity indicators.

Notably, neither years of professional experience nor general clinical exposure to HF patients independently associated with approval once system-level factors were included in the model. These findings indicate that authorization outcomes are primarily driven by classification logic and policy structures, rather than individual clinician expertise.

### 3.8 Qualitative findings: Decision-making at the interface of evidence, prescribing, and authorization

Semi-structured interviews were conducted with a purposive sample of 22 physicians, including 12 treating physicians (general practice, family medicine, and internal medicine) and 10 insurance physicians. Participants were selected to capture variability in prescribing behavior, authorization roles, practice settings, and years of experience. Data saturation was achieved after the 20th interview, with no new themes emerging in the final interviews.

Thematic analysis identified four interrelated themes (Table 8) that elucidate how evidence-based recommendations for SGLT2 inhibitors in HF are translated—or obstructed—within real-world clinical and authorization pathways.

**TABLE 8:** Summary of qualitative themes and illustrative quote**s**.

Theme	Subtheme	Description	Representative illustrative quotes
Perceived boundaries of clinical authority and role legitimacy	Role uncertainty among treating physicians	Treating physicians expressed uncertainty regarding their legitimacy to initiate SGLT2 inhibitors for HF, despite awareness of guideline recommendations	“I know it’s in the guidelines, but if insurance rejects it, it feels like I was not supposed to start it in the first place.” (GP, public sector)
​	Specialty-based legitimacy	Authorization decisions were frequently influenced by prescriber specialty, with cardiologist involvement perceived as conferring clinical legitimacy	“When it comes from a cardiologist, approval is straightforward. From a GP, even with documents, it’s questioned.” (insurance physician)
Drug classification as a cognitive shortcut in authorization decisions	Diabetes-centric drug identity	SGLT2 inhibitors were commonly conceptualized as diabetes-only medications, limiting recognition of HF indications	“In our system, SGLT2 inhibitors are still diabetes drugs. Heart failure is not the default indication.” (insurance physician)
​	Reliance on internal drug lists and codes	Decision-making often depended on internal formularies and ICD coding rather than guideline-based assessment	“We go by the list and the code. There is not time to reinterpret the drug every time.” (insurance physician)
Risk aversion and defensive decision-making	Fear of professional accountability	Both groups described defensive practices driven by fear of blame, scrutiny, or financial responsibility	“If something goes wrong, nobody asks about guidelines—they ask why you approved an expensive drug.” (insurance physician)
​	Therapeutic inertia following rejection	Repeated authorization rejection discouraged future prescribing attempts by treating physicians	“After a few rejections, you just stop trying. It’s not worth the effort.” (family medicine physician)
Structural enablers for alignment with HF guidelines	Need for standardized protocols	Participants emphasized the absence of clear, guideline-aligned protocols recognizing HF as an approved indication	“If there was a written policy saying this is approved for heart failure, most arguments would disappear.” (insurance physician)
​	Bridging mechanisms (education & pharmacist support)	Targeted education and involvement of clinical pharmacists were viewed as facilitators for aligning evidence and authorization decisions	“A pharmacist or a clear guideline summary adapted for insurance work would really help.” (internal medicine physician)

#### 3.8.1 Perceived boundaries of clinical authority and role legitimacy

Both treating physicians and insurance physicians described clear, albeit informal, boundaries regarding who is considered “authorized” to initiate or endorse SGLT2 inhibitors for HF. Many treating physicians expressed uncertainty about whether initiating SGLT2 inhibitors fell within their professional remit, even when they were aware of guideline recommendations.

“I know it’s in the guidelines, but if I start it and insurance rejects it, it feels like I crossed a line that was not meant for me.”

(General practitioner, public sector).

Insurance physicians frequently framed approval decisions in terms of prescriber specialty rather than clinical indication. Cardiologist involvement was viewed as conferring legitimacy, often independent of documentation quality.

“If it comes from a cardiologist, it’s safer to approve. If it’s from a GP, even with the same echo report, it raises questions.”

(Insurance physician).

This theme underscores how hierarchical perceptions of clinical authority shape both prescribing behavior and authorization outcomes, reinforcing specialty-based gatekeeping.

#### 3.8.2 Drug classification as a cognitive shortcut in authorization decisions

A dominant theme among insurance physicians was reliance on drug class identity rather than evolving therapeutic indications. SGLT2 inhibitors were frequently conceptualized as “diabetes drugs,” with HF perceived as a secondary or exceptional indication.

“In our system, SGLT2 inhibitors are still under diabetes. Heart failure is not the first thing that comes to mind.”

(Insurance physician).

Several participants acknowledged awareness of newer evidence but described practical constraints that favored simplified classification schemes during high-volume authorization workflows.

“We handle dozens of approvals a day. You go by the class and the code. You do not have time to re-evaluate the whole drug identity every time.”

This cognitive shortcut explains the persistence of misclassification identified quantitatively and highlights how administrative efficiency can inadvertently conflict with evidence-based care.

#### 3.8.3 Risk aversion, accountability, and defensive decision-making

Risk aversion emerged as a unifying theme across both groups, albeit driven by different concerns. Treating physicians feared professional scrutiny and repeated rejection, while insurance physicians emphasized accountability for financial decisions and adverse outcomes.

“If something goes wrong, nobody will ask about the guideline—they will ask why you approved an expensive drug.”

(Insurance physician).

This defensive orientation often resulted in conservative decision-making, favoring denial or deferral even when clinical justification was strong.

“After two or three rejections, you stop trying. You save the patient and yourself the frustration.”

(Family medicine physician).

Such dynamics contribute to therapeutic inertia and help explain why knowledge and positive attitudes do not consistently translate into action.

#### 3.8.4 Structural enablers for alignment with heart failure guidelines

Despite pervasive barriers, participants identified several enablers that could facilitate alignment between evidence, prescribing, and authorization. These included clear national or insurance-level protocols, shared clinical language between prescribers and reviewers, and formal endorsement of HF indications within drug approval systems.

“If there was a clear protocol saying this drug is approved for heart failure, half of the debate would disappear.”

(Insurance physician).

Both groups also highlighted the potential role of clinical pharmacists and targeted continuing education in bridging interpretation gaps.

“If someone summarized the guideline in a way that fits insurance work, it would change how we decide.”

These insights point toward actionable system-level interventions rather than individual-level blame.

Taken together, the qualitative findings reveal that barriers to SGLT2 inhibitor use in HF are deeply embedded within professional hierarchies, cognitive classification practices, and defensive institutional cultures. Misalignment between evolving pharmacotherapeutic evidence and authorization logic is not solely attributable to lack of knowledge, but rather to structural decision frameworks that prioritize risk containment and administrative efficiency.

### 3.9 Integration of quantitative and qualitative findings (joint display)

To enhance interpretive depth and strengthen explanatory power, quantitative and qualitative findings were integrated using a joint display approach. This integration elucidates how measured patterns in knowledge, attitudes, prescribing behavior, and authorization outcomes are shaped by underlying cognitive, professional, and system-level processes identified through qualitative inquiry.

The joint analysis shown in Table 9 revealed a consistent pattern of misalignment between evidence-based intent and system-level execution. Quantitative findings demonstrated that misclassification of SGLT2 inhibitors as diabetes-only medications and absence of guideline-aligned insurance protocols were the strongest predictors of authorization rejection. Qualitative findings provided explanatory insight into these associations, revealing that authorization decisions are often driven by cognitive shortcuts, role-based legitimacy, and defensive institutional cultures rather than direct appraisal of clinical evidence.

**TABLE 9:** Joint display integrating quantitative and qualitative finding**s**.

Quantitative finding	Supporting quantitative evidence	Qualitative explanation (theme)	Integrated interpretation
Misclassification strongly predicts rejection	Misclassification associated with 82% lower odds of approval (aOR 0.18, p < 0.001)	Drug classification used as cognitive shortcut; SGLT2 inhibitors viewed as “diabetes drugs”	Authorization decisions rely on historical drug identity rather than evolving indications
Knowledge does not guarantee initiation	High knowledge score predicts initiation, but effect attenuated by perceived rejection	Therapeutic inertia after repeated rejection; perceived futility	Structural barriers override clinical competence
Non-cardiologist prescriptions less likely to be approved	Non-cardiologist prescriber aOR 0.44 for approval (p = 0.003)	Specialty-based legitimacy and hierarchical trust	Approval decisions reflect professional hierarchy more than documentation quality
Absence of insurance protocol predicts rejection	Lack of HF protocol aOR 0.31 for approval (p < 0.001)	Reliance on internal lists and ICD codes	Policy void leads to inconsistent, *ad hoc* decision-making
Cost considerations influence approval	Cost flagged reduced approval likelihood (borderline significance)	Defensive accountability and financial scrutiny	Economic pressure shapes conservative authorization behavior
Guideline familiarity not independently predictive	Guideline knowledge not significant in approval model	Guidelines not operationalized in insurance workflow	Evidence awareness without structural integration has limited impact

Importantly, the joint display highlights that physician knowledge and positive attitudes—while necessary—were insufficient to overcome structural barriers embedded within authorization frameworks. Treating physicians with high knowledge scores frequently reported therapeutic inertia following repeated rejection, a behavior that was quantitatively associated with lower initiation rates and qualitatively attributed to frustration, perceived futility, and fear of professional scrutiny.

Similarly, insurance physicians’ reliance on drug-class identity and internal policy constraints explained why guideline familiarity alone did not independently predict approval in multivariable models. This integrated interpretation underscores that access to SGLT2 inhibitors for heart failure is mediated not by isolated decision-makers, but by an interaction between evidence, professional hierarchies, and institutional logics.

## 4 Discussion

This mixed-methods study provides a comprehensive examination of the multifaceted barriers shaping the real-world use of SGLT2 inhibitors for HF in Jordan. By integrating perspectives from treating physicians and insurance physicians, the findings illuminate a critical disconnect between contemporary evidence-based recommendations and the systems governing medication access. Importantly, this disconnect is not primarily driven by lack of awareness among clinicians, but rather by structural and cognitive mechanisms embedded within authorization and reimbursement frameworks.

Quantitative findings indicate that treating physicians demonstrated moderate to high levels of knowledge regarding the role of SGLT2 inhibitors in HF, particularly HFrEF, and generally expressed favorable attitudes toward their use. These findings align with international studies showing growing clinician awareness of SGLT2 inhibitors’ cardiovascular benefits following landmark trials such as DAPA-HF and EMPEROR-Reduced (McMurray et al., 2019; Pac et al., 2020). However, despite this awareness, initiation rates in routine practice remained modest, and authorization approval rates were substantially lower when prescriptions were written for HF in the absence of diabetes mellitus. This divergence reinforces a central insight of the present study: knowledge and positive attitudes, while necessary, are insufficient for implementation when prescribing intent and reimbursement approval are institutionally decoupled.

A key contribution of this study is the identification of diagnostic misclassification of SGLT2 inhibitors as diabetes-only medications as the dominant predictor of authorization rejection. Multivariable analyses demonstrated that misclassification was associated with an over 80% reduction in approval likelihood, exerting a stronger effect than prescriber experience, documentation quality, or guideline familiarity. This finding extends existing literature by shifting explanatory emphasis away from individual clinician behavior toward system-level classification logic as a critical determinant of access. Prior studies examining barriers to SGLT2 inhibitor uptake have often focused on clinician concerns regarding safety, adverse effects, or clinical uncertainty (Greene and Pierce, 2023; Pradhan et al., 2024) In contrast, our findings suggest that in the Jordanian context, rejection is driven primarily by how drugs are cognitively and administratively categorized within authorization systems rather than by prescriber reluctance.

This misclassification phenomenon reflects a broader issue of digital infrastructure lag, whereby reimbursement and authorization software fails to keep pace with evolving pharmacotherapeutic evidence. Participants consistently described automated rejection pathways based on historical drug–indication mapping, in which SGLT2 inhibitors remain anchored to their original antidiabetic identity despite strong guideline endorsement for HF across the ejection fraction spectrum (Heidenreich et al., 2022; McDonagh et al., 2021). Similar forms of digital lag have been described in other settings, where outdated formularies, ICD-based algorithms, and step-therapy rules act as *de facto* gatekeepers to care, constraining access independently of clinician intent (Tummalapalli et al., 2021).

Our findings share notable similarities with international studies yet reveal important context-specific distinctions. Real-world surveys and qualitative work in the United States (Greene and Pierce, 2023; Pradhan et al., 2024), Canada (Yi et al., 2024), and Australia/New Zealand (Teng et al., 2024) consistently identify clinical inertia, high out-of-pocket costs, prior-authorization requirements, formulary restrictions, and the perception of SGLT2 inhibitors as “diabetes-only” medications as key barriers to uptake in heart failure. Recent US analyses further demonstrate that prior-authorization mandates for SGLT2 inhibitors in heart failure are associated with substantially delayed filling (6.75-fold longer time to fill) and more than doubled non-fill rates (Mukhopadhyay et al., 2026). However, our study is unique in three respects. First, it is the first to systematically capture the perspectives of insurance physicians, the formal gatekeepers in Jordan’s mixed public-private system, revealing that misclassification of SGLT2 inhibitors as diabetes-only agents (61.2% prevalence) is the strongest independent predictor of rejection (adjusted OR 0.18). Second, unlike high-income settings where guideline integration into formularies has advanced and generic availability is emerging, Jordan exemplifies persistent digital and policy lag in authorization algorithms, which disproportionately amplifies specialty hierarchies and overrides treating physicians’ knowledge. Third, the mixed-methods design with joint-display integration of treating and insurance viewpoints provides explanatory depth rarely achieved in clinician-only surveys conducted elsewhere. These differences underscore how systemic authorization barriers in middle-income countries may be more entrenched than the predominantly clinician- or patient-level barriers documented in high-resource settings.

Although this study did not collect primary pharmacoepidemiologic data, the low initiation and approval rates observed in our cohort are consistent with the very low utilization of SGLT2 inhibitors reported in the Jordanian Heart Failure Registry (JoHFR), where these agents were prescribed in only 9% of patients with heart failure (Abu-Hantash et al., 2025; Izraiq et al., 2024) Future pharmacoepidemiologic studies using national claims or prescription databases would be valuable to quantify indication-specific prescribing patterns and to evaluate the downstream impact of the authorization barriers identified in the present study.

The qualitative findings provide essential explanatory depth to these statistical associations. Insurance physicians described reliance on drug-class identity as a pragmatic response to high-volume authorization demands, limited time for individualized clinical appraisal, and accountability pressures related to cost containment. In this context, SGLT2 inhibitors continue to be processed through a diabetes-centric lens, despite their evolving pharmacological role in cardiovascular disease. This phenomenon illustrates how administrative efficiency, while operationally necessary, may inadvertently perpetuate outdated therapeutic paradigms. Understanding why such administrative logic persists requires attention to the organizational structure of authorization systems. The finding that all insurance physicians were general practitioners is not a sampling artifact but reflects the administrative design of authorization systems in the local context. Authorization roles prioritize policy adherence and cost control over specialty expertise, effectively positioning generalist physicians as gatekeepers for highly specialized pharmacotherapy decisions. This structural arrangement amplifies reliance on algorithmic and protocol-driven decision frameworks rather than specialist clinical judgment.

Another salient finding is the role of professional hierarchy and role legitimacy in shaping both prescribing and approval decisions. Prescriptions initiated by cardiologists were consistently more likely to be approved, even after adjustment for clinical and documentation factors. Qualitative narratives revealed that cardiologist endorsement functions as a proxy for clinical legitimacy, effectively mitigating perceived risk for insurance decision-makers. Conversely, general practitioners—despite being legally authorized to prescribe—often perceived initiation of SGLT2 inhibitors as exceeding their professional remit. This dynamic reinforces therapeutic inertia and perpetuates a specialist-centric model of HF care, echoing findings from studies in Europe and Australia that document lower uptake of guideline-directed HF therapies outside cardiology-led settings (Greene and Pierce, 2023; Teng et al., 2024).

While international literature consistently demonstrates suboptimal uptake of SGLT2 inhibitors in HF, particularly in primary care and non-specialist contexts, the present study extends this evidence by explicitly interrogating the authorization interface. This dimension remains underexplored in both high-income and middle-income countries. In Jordan, where private insurance plays a substantial role in healthcare financing, insurance physicians emerge as critical gatekeepers whose decision frameworks materially shape patient access to evidence-based therapy. The absence of standardized, guideline-aligned authorization protocols exacerbates variability in approval decisions and places undue emphasis on prescriber specialty and diagnostic coding rather than therapeutic indication.

Regression analyses further elucidated the asymmetry between determinants of initiation and determinants of approval. While initiation by treating physicians was associated with knowledge, attitudes, and recent HF-related training, consistent with prior implementation studies (Hutcheson et al., 2018), HF approval was overwhelmingly governed by insurance-level factors, particularly misclassification practices and lack of formal recognition of HF as an approved indication. This asymmetry creates a systemic bottleneck in which evidence-based prescribing intent is filtered through non-clinical criteria, undermining the translation of robust trial evidence into population-level benefit.

From a pharmacological perspective, these findings highlight a critical challenge in the life cycle of drug indications where regulatory approval and guideline endorsement do not automatically translate into operational recognition within reimbursement systems. As drug classes evolve and acquire new indications, lagging updates in formularies, coding systems, and authorization criteria can create persistent access barriers. In the case of SGLT2 inhibitors, failure to recalibrate these systems risks limiting realization of the cardiovascular and renal benefits demonstrated in large randomized trials and meta-analyses (McGuire et al., 2021).

The findings of this study have important implications for health policy, reimbursement frameworks, and everyday clinical practice in Jordan and similar healthcare systems. Most notably, they demonstrate that gaps in access to evidence-based heart failure pharmacotherapy are not primarily attributable to deficiencies in clinician knowledge or unwillingness to prescribe, but rather to structural misalignment between contemporary clinical evidence and insurance authorization mechanisms. Addressing this misalignment requires interventions that operate at the level of policy design and institutional processes, rather than relying solely on individual clinician education.

At the policy level, the persistence of misclassification of SGLT2 inhibitors as diabetes-only medications highlights the urgent need for formal, guideline-aligned recognition of heart failure as a primary reimbursable indication. Insurance formularies, internal drug lists, and authorization algorithms must be updated to reflect the current pharmacological role of SGLT2 inhibitors across the heart failure spectrum, independent of diabetes status. Without such formal recognition, authorization decisions will continue to rely on outdated drug identities and diagnostic shortcuts, perpetuating inequities in access despite strong clinical evidence.

The study also underscores the importance of standardizing authorization criteria across insurance providers. The observed variability in approval decisions, driven by prescriber specialty, documentation interpretation, and informal heuristics, suggests that absence of explicit protocols allows discretionary practices to dominate. Development of transparent, nationally harmonized authorization frameworks aligned with international heart failure guidelines could reduce unwarranted variability, enhance predictability for clinicians, and limit delays in treatment initiation. Such frameworks would also protect insurance physicians by shifting decisions from individual discretion to institutionally endorsed standards.

From a practice perspective, the findings challenge the implicit assumption that specialist endorsement should function as a prerequisite for access to advanced heart failure pharmacotherapy. While cardiologist involvement remains essential for complex cases, over-reliance on specialty-based legitimacy may inadvertently marginalize general practitioners and family physicians who are often responsible for longitudinal heart failure care. Clear delineation of prescribing authority, supported by guideline-based protocols, could empower non-cardiologist physicians to initiate SGLT2 inhibitors appropriately while maintaining accountability and safety.

The results further suggest that educational interventions should be reoriented to include insurance physicians as a key target audience. Traditional continuing medical education efforts have largely focused on prescribers, yet this study demonstrates that authorization decisions are frequently made by physicians whose clinical exposure to heart failure is indirect and whose decision-making frameworks are shaped by policy and cost considerations. Tailored educational initiatives that translate guideline recommendations into authorization-relevant criteria—rather than purely clinical narratives—may be more effective in aligning approval practices with evidence-based care.

In addition, the findings point to a potential role for clinical pharmacists as intermediaries within fragmented pharmacotherapy pathways. Pharmacists, particularly those with training in cardiovascular pharmacotherapy, are well positioned to support documentation standardization, clarify indications, and facilitate communication between prescribers and insurers. Integrating pharmacist-led medication review or authorization support services could reduce administrative friction and mitigate the deterrent effects of repeated rejection experienced by treating physicians. This recommendation is supported by local interventional evidence from Jordan demonstrating that pharmacist-led heart failure management programs can improve patient outcomes, underscoring the feasibility of integrating pharmacists into complex medication pathways (Zakareia et al., 2024).

In sum, improving access to SGLT2 inhibitors for heart failure in Jordan will require coordinated action that bridges clinical evidence, prescribing authority, and reimbursement policy. Interventions that address misclassification, standardize authorization processes, and integrate all decision-making stakeholders into guideline implementation efforts are likely to yield greater impact than isolated educational or behavioral approaches. By reframing access barriers as systemic rather than individual failures, this study provides a foundation for policy reforms that support equitable and evidence-based heart failure care.

## 5 Limitations

Several limitations of this study should be acknowledged when interpreting the findings. First, the quantitative component employed a convenience sampling strategy, which may limit the generalizability of results to all physicians involved in heart failure management and medication authorization in Jordan, and selection bias cannot be fully excluded. Although efforts were made to include participants from diverse practice settings and authorization roles, the cohort cannot be considered fully representative of the national physician workforce. National demographic data describing the distribution of physician characteristics across specialties, age groups, and practice settings are limited, making direct comparisons with the broader physician population challenging. Nevertheless, the inclusion of both treating physicians and insurance physicians provides a broad range of perspectives relevant to real-world prescribing and authorization processes. As such, the findings offer meaningful insights into practical barriers to SGLT2 inhibitor prescribing and coverage decisions, although they should be interpreted with appropriate caution regarding generalizability.

Second, the study relied on self-reported data regarding prescribing behavior, authorization decisions, and perceived barriers. Such data are inherently subject to recall bias and social desirability bias, particularly in relation to practices that may be viewed as non-aligned with clinical guidelines. However, the consistency observed between quantitative findings and qualitative narratives, as well as the convergence of perspectives across physician groups, suggests that the reported patterns reflect genuine systemic phenomena rather than isolated reporting artifacts.

Third, although the questionnaire underwent rigorous development and validation processes, the knowledge and attitude measures were based on a study-specific instrument rather than a previously standardized scale. While content validity and internal consistency were robust, direct comparison of absolute knowledge or attitude scores with those reported in other settings may be limited. Future studies may benefit from further psychometric evaluation or cross-national validation of similar instruments.

Fourth, the qualitative component was conducted in two languages, with interviews undertaken in Arabic or English according to participant preference. Despite careful translation, back-review, and consensus-based resolution of discrepancies, some nuances of language, emphasis, or cultural context may have been partially attenuated in translation. However, the use of bilingual researchers with clinical expertise and systematic cross-checking procedures mitigates this risk and supports the credibility of the qualitative findings.

Fifth, the qualitative sample size, while sufficient to achieve thematic saturation, was relatively small and focused on physicians directly involved in prescribing and authorization. Perspectives of other stakeholders, such as pharmacists, hospital administrators, or patients, were not included. Their inclusion in future research could provide a more comprehensive understanding of how authorization processes affect medication access and continuity of care.

Sixth, the study did not include objective verification of authorization outcomes through insurance claims data or pharmacy dispensing records. As a result, approval and rejection rates were based on physician reports rather than administrative datasets. While such linkage was beyond the scope of the current study, future work integrating survey data with claims or dispensing records could provide more granular insight into authorization dynamics and their clinical consequences.

Finally, this study was conducted within the specific regulatory, insurance, and healthcare context of Jordan. Although many of the identified mechanisms, such as misclassification of drug indications, reliance on prescriber specialty, and cost-driven authorization frameworks, are likely applicable to other middle-income settings, caution is warranted when extrapolating findings to healthcare systems with different financing and regulatory structures.

Despite these limitations, the study offers a rigorous and novel examination of the intersection between evidence, prescribing, and authorization for SGLT2 inhibitors in heart failure. The mixed-methods design, integration of quantitative and qualitative data, and focus on system-level mechanisms provide a robust foundation for informing policy and practice in Jordan and similar healthcare contexts.

## Data Availability

The original contributions presented in the study are included in the article/Supplementary Material, further inquiries can be directed to the corresponding author.
