# Supplementary material for: Barriers to prescribing and insurance approval of SGLT2 inhibitors for heart failure: a mixed-methods study in Jordan
Source: Front Pharmacol. 2026 Apr 7;17:1803731. doi: 10.3389/fphar.2026.1803731 (PMC13095551; doi:10.3389/fphar.2026.1803731)
Supplement: Supplementary file 1 [file Table1.docx]

**Supplementary Appendix I (Version date June, 2025)**

This self-administered questionnaire was developed to assess physician knowledge, attitudes, prescribing behaviors, and perceived barriers to SGLT2 inhibitor use in heart failure. Items were informed by current ESC/ACC guidelines and the Capability–Opportunity–Motivation–Behavior (COM-B) model. The tool was piloted for clarity and content validity among a small group of cardiologists and primary care physicians (n=8) prior to full deployment. The questionnaire was administered anonymously online via Google Forms. Estimated completion time: 10–12 minutes.

**Section 1 — Physician and Practice Characteristics**

1. Age (years): ______
2. Gender:
   □ Male
   □ Female
3. Primary specialty:
   □ General Practice
   □ Family Medicine
   □ Internal Medicine
4. Years since medical graduation: ______
5. Primary work sector:
   □ Public (Ministry of Health / Royal Medical Services / University hospitals)
   □ Private
6. Main practice setting:
   □ Hospital-based
   □ Outpatient clinic
   □ Insurance authorization unit
7. Average number of heart failure patients managed per month:
   □ <5
   □ 5–10
   □ 11–20
   □ >20
8. Have you received formal continuing medical education (CME) on contemporary heart failure pharmacotherapy within the past 3 years?
   □ Yes
   □ No
9. Do you routinely consult international heart failure guidelines (ESC, ACC/AHA)?
   □ Yes
   □ No
10. Do you have legal prescribing authority for SGLT2 inhibitors within your institution?
    □ Yes
    □ No
    □ Unsure

**For insurance physicians only:**
11. Do you routinely review authorization requests for high-cost cardiovascular medications?
□ Yes
□ No

**Section 2 — Knowledge of SGLT2 Inhibitors in Heart Failure**

Please select the single best answer.

12. SGLT2 inhibitors reduce heart failure hospitalizations independent of diabetes status.
□ True
□ False
□ Unsure

13. Current international guidelines recommend SGLT2 inhibitors as foundational therapy for patients with heart failure with reduced ejection fraction (HFrEF).
□ True
□ False
□ Unsure

14. Evidence supports the use of SGLT2 inhibitors in selected patients with heart failure with preserved ejection fraction (HFpEF).
□ True
□ False
□ Unsure

15. SGLT2 inhibitors should only be prescribed to patients with diabetes.
□ True
□ False
□ Unsure

16. At approximately which estimated glomerular filtration rate (eGFR) can SGLT2 inhibitors generally be initiated safely in heart failure patients?
□ ≥20–25 mL/min/1.73m²
□ ≥45 mL/min/1.73m²
□ ≥60 mL/min/1.73m²
□ Contraindicated in chronic kidney disease

17. Which of the following represents a major clinical benefit associated with SGLT2 inhibitors?
□ Reduction in HF hospitalization
□ Immediate improvement in ejection fraction
□ Elimination of diuretic need
□ Cure of underlying cardiomyopathy

18. Have major trials such as DAPA-HF or EMPEROR demonstrated cardiovascular benefit in non-diabetic HF populations?
□ Yes
□ No
□ Unsure

19. Which adverse effect requires clinical monitoring when initiating SGLT2 inhibitors?
□ Volume depletion
□ Severe hepatotoxicity
□ Pulmonary fibrosis
□ Hypercalcemia

20. SGLT2 inhibitors are considered part of guideline-directed medical therapy (GDMT) for HF.
□ True
□ False
□ Unsure

21. Early initiation of SGLT2 inhibitors is associated with improved clinical outcomes in HF.
□ True
□ False
□ Unsure

**Section 3 — Attitudes Toward SGLT2 Inhibitors**

**Response scale:**
1 = Strongly disagree
2 = Disagree
3 = Neutral
4 = Agree
5 = Strongly agree

22. SGLT2 inhibitors should be initiated early as part of guideline-directed therapy for heart failure.

23. I feel confident initiating or approving SGLT2 inhibitors for HF without cardiology consultation.

24. SGLT2 inhibitors represent standard therapy for HF irrespective of diabetes status.

25. Prescribing SGLT2 inhibitors for HF falls outside the drug’s traditional therapeutic role.

26. Initiation of SGLT2 inhibitors should be restricted to cardiologists.

27. I am hesitant to initiate or approve SGLT2 inhibitors due to potential insurance rejection.

28. The clinical benefits of SGLT2 inhibitors outweigh their risks in most HF patients.

29. Authorization decisions are strongly influenced by institutional policies and cost considerations.

30. I feel professionally supported when prescribing evidence-based HF therapies.

31. Medico-legal concerns influence my decision to prescribe or approve newer HF therapies.

32. I believe non-specialist physicians should be empowered to initiate guideline-directed HF treatments.

33. Administrative complexity discourages optimal prescribing.

**Section 4 — Real-World Prescribing and Authorization Practices**

34. In the past 12 months, have you prescribed or attempted to prescribe an SGLT2 inhibitor for heart failure?
□ Yes
□ No

35. How frequently are SGLT2 inhibitor prescriptions for HF (without diabetes) approved by insurance providers?
□ Always
□ Usually
□ Sometimes
□ Rarely
□ Never

36. Have you ever experienced rejection of an SGLT2 inhibitor prescription due to classification as a diabetes-only medication?
□ Yes
□ No
□ Unsure

37. Does prescriber specialty influence approval likelihood?
□ Yes
□ No
□ Unsure

If yes: Which specialty is most likely to receive approval?
□ Cardiologist
□ Internal medicine
□ GP/Family physician
□ Endocrinologist
□ Other: ______

38. When prescriptions are rejected, how often are they successfully approved after appeal?
□ Often
□ Sometimes
□ Rarely
□ Never
□ Not applicable

**Section 5 — System-Level Barriers and Authorization Environment**

**Likert scale (1–5).**

39. Misclassification of SGLT2 inhibitors as diabetes-only drugs is a common reason for authorization rejection.

40. Lack of guideline-aligned insurance protocols contributes to inconsistent approval decisions.

41. Documentation requirements create barriers to timely therapy initiation.

42. Cost-containment pressures influence authorization outcomes.

43. Automated approval systems do not adequately reflect evolving clinical evidence.

44. Clear national reimbursement policies would improve access to guideline-directed HF therapies.

45. Greater collaboration between prescribers and insurers could reduce unnecessary rejection.

46. Updating formularies in response to emerging evidence is essential for improving patient care.
